# Will 14-3-3*η* Be a New Diagnostic and Prognostic Biomarker in Rheumatoid Arthritis? A Prospective Study of Its Utility in Early Diagnosis and Response to Treatment

**DOI:** 10.1155/2022/1497748

**Published:** 2022-01-04

**Authors:** Doaa Shawky Alashkar, Radwa Mostafa Elkhouly, Amira Yousef Abd Elnaby, Doaa Waseem Nada

**Affiliations:** ^1^Physical Medicine, Rheumatology and Rehabilitation, Faculty of Medicine, Tanta University, Tanta, Egypt; ^2^Clinical Pathology, Faculty of Medicine, Tanta University, Tanta, Egypt

## Abstract

**Results:**

Serum14-3-3*η* levels were significantly higher in all RA patients than in controls (*P* < 0.001), its sensitivity was 86.7% and 88.3% in early and established RA patients with a significant difference with RF and ACCP at early disease, and the specificity was 96.7%. There was a significant reduction of 14-3-3*η* levels 6 months after treatment in the first group (*p*=0.004), and there was a significant positive correlation between serum 14-3-3*η* levels and parameters of disease activity and severity.

**Conclusion:**

14-3-3*η* could be a novel, potent, and efficacious diagnostic, and prognostic marker for RA with high sensitivity, that may become a new therapeutic target for RA.

## 1. Introduction

Rheumatoid arthritis (RA) is a chronic autoimmune disease that affects about 1.5% of the population. There are multiple pathophysiological factors in its etiology and manifestations, and there is high heterogeneity among patients throughout the disease. If untreated, RA results in severe joint destruction, leading to impaired physical activity and disability [1]. It is now understood that the disease outcome and patient prognosis can be significantly improved by early identification of RA and prediction of the disease severity at diagnosis for the implementation of an effective treatment strategy [2].

However, early diagnosis of RA is difficult due to limited clinical symptoms and an absence of physical signs. Laboratory data of patients with arthralgia, such as levels of rheumatoid factor (RF) and anticyclic citrullinated peptide (ACCP), are not remarkable for RA. Therefore, there is a great need for the development and validation of new biomarkers that have predictive capacity for RA [3].

RF is an autoantibody that targets the Fc region of IgG. The sensitivity of RF testing is 60 to 86% for established RA and 57% for early RA, but the utility of RF testing is somewhat limited by its low specificity of 70 to 85% for established and early RA [4]. Recently, in 2010, cyclic citrullinated peptide (CCP) was added to the classification criteria for RA of the American College of Rheumatology/European League Against Rheumatism (ACR/EULAR) [5]. CCP testing has sensitivity similar to that of RF for established RA (64% to 88%) and early RA (59%) [6], but its specificity (90 to 99%) is higher for both established and early disease [7].

Although CCP is more specific than RF testing, it is not considered a replacement for RF. Serology tests for both markers are included in the ACR/EULAR classification criteria for RA [5], and studies show that the combined use of the markers provides greater sensitivity. However, 28–44% of patients with early RA experience negative test results, and patients who develop erosive RA may remain negative for both markers. Thus, other markers for RA have been investigated [8].

14-3-3 proteins represent a family of intracellular chaperonins that are exclusively expressed in eukaryotic cells. Seven isoforms that share more than 50% amino acid homology have been isolated: beta (*β*), epsilon (*ε*), gamma (*γ*), eta (*η*), tau (*τ*), zeta (*ζ*), and sigma (*σ*). The 14-3-3 family can interact with more than 200 intracellular proteins [9]. Thus, it is involved in coordinating an array of biological processes, including protein trafficking, signaling, and cytoskeletal transport [10].

14-3-3*η* protein represents a novel biomarker for the detection of RA. It may play a role in stimulating tumor necrosis factor-alpha, metalloproteinases, and other inflammatory mediators that are critical to joint erosion. This demonstrates its prognostic value with aims for treatment. Blood levels of 14-3-3*η* tend to be elevated in patients with RA, but not in those with other collagen diseases [11]. One of the advantages of 14-3-3 *η* as an RA marker is that it can improve the identification rates of early RA. Maksymowych et al. found that adding 14-3-3*η* to RF and CCP antibody testing increased diagnostic sensitivity for early RA patients [11].

This study aims to evaluate the role of 14-3-3*η* in the early diagnosis of RA, as well as its sensitivity and specificity relative to traditional biomarkers. Furthermore, we defined the correlation of 14-3-3*η* with the disease's activity, severity, and ultrasonographic features from RA patients with established disease. Lastly, we clarify the effect of anti-TNF therapy (etanercept) on 14-3-3*η* levels in early RA patients.

## 2. Subjects and Method

**Study population:** 80 patients early diagnosed to have RA (group I), 80 patients with established disease (group II), and 80 age- and sex-matched healthy volunteers (group III) were included in the study. All patients were selected from Physical Medicine, Rheumatology, and Rehabilitation Clinics, Tanta University Hospitals. The ethical approval was obtained from the hospital ethical research committee of the Faculty of Medicine at Tanta University, approval code: 32768/12/20. All investigations were explained to all patients, **each patient entering the study was informed and gave written informed consent,** and the trial was conducted according to the Declaration of Helsinki principles.

RA patients were diagnosed according to the American College of Rheumatology (ACR)/European League Against Rheumatism (EULAR) 2010 Criteria for RA [5], and patients with other autoimmune diseases, infections, osteoporosis, and malignancy were excluded.

### 2.1. Methods

#### 2.1.1. Group I (Early RA ≤ 6 Months)

Laboratory assessment of RF, ACCP, and 14-3-3*η* serum levels was conducted to calculate the sensitivity and specificity of each at early disease onset. Serum levels of 14-3-3*η* were reassessed 6 months after anti-TNF therapy (etanercept; Enbrel with a dose of 50 mg/week SC).

Laboratory assessment of serum 14-3-3*η* level: venous blood samples were collected and centrifuged at 1000 g for 15 min for serum separation. The serum samples were frozen at −70°C until used for assay of serum 14-3-3*η* level using the Human 14-3-3 Protein (14-3-3 Pro) ELISA Kit Cat No. MBS269690 using the double-sandwich ELISA technique. The precoated antibody is a human 14-3-3 promonoclonal antibody, and the detecting antibody is a polyclonal antibody with biotin labeled. Samples and biotin labeling antibodies are added into ELISA plate wells and washed out with PBS or TBS. Then Avidin-peroxidase conjugates are added to ELISA wells in order; TMB substrate was used for coloring after the reactant is thoroughly washed out by PBS or TBS. TMB turns into blue in peroxidase catalytic and finally turns into yellow under the action of acid. The color depth and the testing factors in samples are positively correlated [12].

#### 2.1.2. Group II: Clinical and Laboratory Assessment

Full medical history taking and general and locomotor system examination: assessment of activity by Disease Activity Score 28 (DAS-28):

DAS-28 = 0.56√ (TEN28) + 0.28√ (SW28) + 0.70 Ln (ESR) + 0.014 [13]. Assessment of severity by the rheumatoid arthritis severity scale (RASS) that consists of three scales (disease activity, functional impairment, and physical damage) was conducted using a range of 1–100, with a score of 1 meaning there is no evidence of the condition, and 100 means maximum level of progression [14]. Serum levels of RF, ACCP, and 14-3-3*η* were assessed to determine their sensitivity and specificity in patients with established disease to be compared with those in patients at early disease onset and laboratory assessment of ESR and CRP.

Radiographic assessment: plain X-ray of both hands and wrists was performed, sixteen joints were evaluated in each hand using the modified Larsen score MLS 1995, and the final score ranges from 0 to 160 [15].

Ultrasonographic assessment: systematic multiplanar gray-scale ultrasound (GSUS) and power Doppler ultrasound (PDUS) examinations were performed on the most clinically affected wrist joint, in a standardized manner based on the guidelines of the European League Against Rheumatism (EULAR) using joint inflammation and damage parameters (synovial thickening, joint effusion, bone erosion, and power Doppler PD activity): **(1) synovial thickening:** nondisplaceable and poorly compressible visualized abnormal hypoechoic intra-articular tissue, in longitudinal and transverse planes, measured in millimeters, **2) joint effusion:** a compressible anechoic intracapsular area that is semiquantitatively examined as follows: **grade 0:** no effusion; **grade 1:** minimal; **grade 2:** moderate (without distension of the joint capsule); and **grade 3:** extensive (with distension of the joint capsule), **3) vascularity by Power Doppler (PD):** semiquantitative grading of the PD was assessed as follows: **grade 0**: no flow in the synovium; **grade 1**: single vessel signal; **grade 2**: less than half of the area of the synovium is filled with vessel signal; and **grade 3**: more than half of the area is filled with vessel signal, and **4) bone erosion score:** an interruption of the bone surface on two perpendicular planes defined as erosion, assessed as follows: **grade 0:** normal bone surface; **grade 1:** bone surface irregularity without defect in two planes; **grade 2:** surface defect in two planes; and **grade 3:** bone defect creating extensive bone destruction [16, 17].

**Statistical analysis** was carried out using the statistical package for social sciences (SPSS) software, version 16.0 for Windows (SPSS Inc., Chicago, Illinois, USA). Demographic data between patients and controls were compared using chi-square and unpaired Student's *t*-tests. Pearson's correlation coefficient (*r*) was used to determine the correlations. Data are expressed as a mean ± SD. *P* values of less than 0.05 were considered statistically significant and less than 0.001 were considered highly significant for differences and correlations.

## 3. Results

The results of this study revealed a highly significant difference between 14-3-3*η*, RF, and ACCP regarding sensitivity for RA diagnosis at early disease onset (*p* < 0.001), but not in established disease. The sensitivity of 14-3-3*η* was 86.7% and 88.3% in early and established RA, respectively. Serum levels of 14-3-3*η* were highly significant in all RA patients compared to controls (*p* < 0.001), and there was a significant reduction at 6 months after anti-TNF therapy in patients with early disease (*p*=0.004) (Table 1).

**Figure 1:**
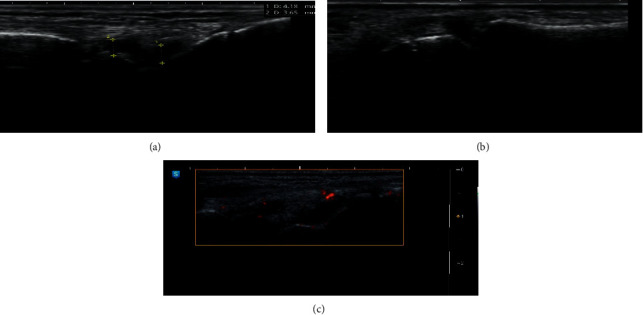
(a) Ultrasonographic examination of the wrist of an RA patient showing synovial thickness: at radiocarpal = 4.18 mm and intercarpal = 3.65 mm, (b) ultrasonographic examination of the wrist of an RA patient showing grade 2 carpal bone erosion, and (c) ultrasonographic examination of the wrist of an RA patient showing grade 2 Doppler activity.

Regarding parameters of active disease in patients with established RA, the median DAS was 4.13, and the synovial thickness of the most affected wrist was 2.85 mm according to ultrasound. Most patients showed grade 2 joint effusion and Doppler activity. Assessment of disease severity in these patients revealed a median of Modified Larsen Score (MLS) of 34 and RASS of 70, with 31.7% showing grade 2 bone erosions in 31.7% of them (Figure 1 and Table 2).

**Figure 2:**
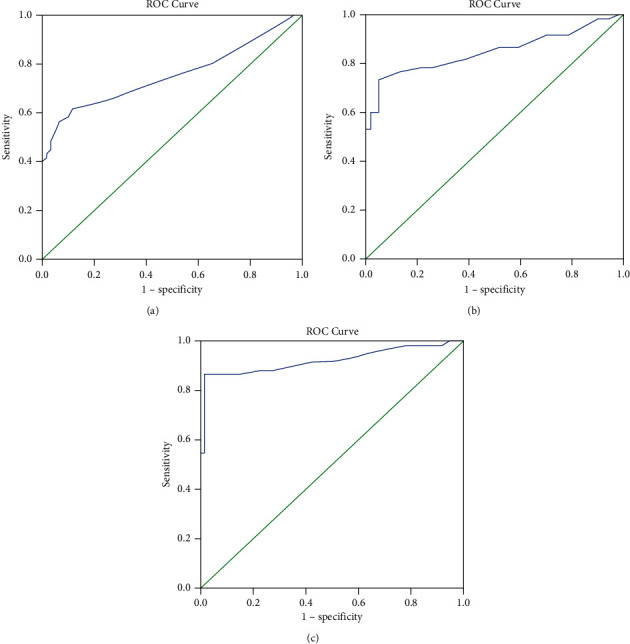
Receiver operating characteristic ROC curve analysis for (a) RF, (b) anti-CCP, and (c) 14-3-3*η*.

14-3-3*η* showed a significant positive correlation with clinical, laboratory, and radiological parameters of disease activity and severity in patients with established disease (Table 3). ROC curve analysis yielded sensitivities of 61.7%, 68.3%, and 86.7% for RF, ACCP, and 14-3-3*η* at early disease onset at cutoff values of 7.93, 21.20, and 0.23 with a specificity of 88.3%, 95%, and 96.7%, respectively (Figure 2 and Table 4).

## 4. Discussion

Early diagnosis of RA is now a matter of interest to prevent joint damage and improve clinical and functional outcomes. Seronegative of RF and ACCP at early disease stages necessitates a critical need to identify new biomarkers with high diagnostic potential at early disease onset [18,19].

Seven isoforms of the 14-3-3 protein family have been isolated, and one of these is the 14-3-3*η* protein. This isoform is highly expressed extracellularly in the joints of patients with erosive RA and strongly correlated with MMP-1 and MMP3 in both synovial fluid and serum. This characterizes its biological expression and pathogenic role in rheumatologic disease processes [20]. A study of synovial fluid and serum from patients with inflammatory arthritis revealed the presence of two isoforms of 14-3-3, *η* and *η*, with predominance of the latter isoform based on mass spectrometry data [21].

14-3-3 proteins have been described as a key component of exosomes, so their extracellular expression in RA is believed to be mediated in part through an exosomal process. Externalization of 14-3-3 proteins occurs as a result of an active secretory mechanism in inflammation, which is suggested by their release from activated immune cells, including dendritic cells, *T* cells, *B* cells, macrophages, and epithelial cells [22]. Thus, this study was designed to evaluate the diagnostic utility of 14-3-3*η* protein in patients with early RA compared to the traditional biomarkers RF and ACCP, its role in disease pathogenesis, and hence, its theragnostic utility. In addition, we evaluated its prognostic role in disease progression in patients with established RA.

The sensitivities of RF in patients with early and established RA were 61.7% and 78.3%, respectively, and the difference between them was significant. Furthermore, there was a significant difference between the sensitivity of ACCP between the two groups (68.3–85%). In contrast, the difference between 14-3-3*η* in patients of both groups was insignificant (86.7–88.3%). The difference between14-3-3*η*, RF, and ACCP was highly significant in patients with early RA, indicating its high diagnostic utility at early disease stages compared to other biomarkers.

At early disease stages, the specificities of RF, ACCP, and 14-3-3*η* were 88.3%, 95%, and 96.7%, respectively. Furthermore, our results revealed that 36.7% of patients in the first group with negative RF results and 28.8% with negative ACCP results were positive for 14-3-3*η*. This finding draws attention to the value of 14-3-3*η* in the early identification of RA.

ROC curve analysis for RF, ACCP, and 14-3-3*η* at early disease onset yielded a significant difference in the results regarding sensitivity with cutoff values of >7.93, >21.20, and >0.234, respectively. The diagnostic utility of 14-3-3*η* in early disease onset for effective treatment strategies and better outcomes in RA was also assessed by Maksymowych et al. in patients with a median disease duration of <3.5 months. At a cutoff of ≥0.19 ng/mL, the ROC curve yielded a sensitivity of 63.6% and a specificity of 92.6%. The incremental benefit of adding 14-3-3*η* to ACCP and RF resulted in an identification rate of 72% compared to 59% for ACCP alone, and diagnostic capture was increased from 59–72% to 78% when 14-3-3*η* was added to RF [11].

In a study conducted on 619 subjects, the ROC curve analysis demonstrated a significant area under the curve (AUC) of 0.89 with a cutoff of ≥0.19 ng/mL, yielding 77.0% sensitivity and 92.6% specificity [23].

In a study by Maksymowych et al., the ROC curve of 14-3- 3*η* autoantibodies for early RA versus healthy and disease-relevant controls yielded a sensitivity of 73% and a specificity of 79%. Unlike 14-3-3*η* protein, no significant correlation was observed with RF or ACPA. Also, no significant correlation was observed between titers of 14-3-3*η* protein and its autoantibodies. However, their combined expression identified more than 90% of early RA patients [24]. While these data support the complementary aspects of the 14-3-3*η* biomarker platform, further validation according to the OMERACT framework is necessary before 14-3-3*η* biomarkers attain the status of a monitoring marker [24].

In the current study, serum levels of 14-3-3*η* in patients of both RA groups were significantly higher compared to controls. Similarly, the median 14-3-3*η* levels in early RA patients were significantly higher than in disease-relevant controls (0.76 vs. 0.02 ng/mL) in the study by Maksymowych et al. [11]. These levels were three to five times higher than the corresponding levels in the serum of matched donors [21].

In another study, 619 subjects including healthy and disease controls (with other arthropathies, connective tissue disorders, and autoimmune diseases) were assessed for serum 14-3-3*η*. The median 14-3-3*η* concentrations were significantly higher in established RA patients versus healthy individuals (1.12 vs. 0.00 ng/mL) and controls (0.02 ng/mL) [22]. Regarding the diagnostic utility of 14-3-3*η* in undifferentiated RA, 148 patients with arthralgia were recruited in a cohort study to determine whether it was associated with the development of RA. The results revealed that the median 14-3-3*η* levels were significantly higher in the arthralgia group that developed RA, while the 14-3-3*η* and ACCP titers (but not titers of RF) were associated with the development of RA with LRs of 4.12 and 4.16 [23].

To determine the pathogenic role of 14-3-3*η*, *in vitro* cell stimulation studies were performed using human recombinant 14-3-3*η* at concentrations similar to that found in RA patients' sera. The studies revealed that 14-3-3*η* preferentially stimulates cells of the innate immune system, leading to activation of key signaling cascades such as the MAPK/ERK, SAPK/JNK, and the JAK-STAT pathway, which regulate the production of inflammatory and degradative factors responsible for joint damage. Furthermore, several RA-relevant transcripts were upregulated by 14-3-3*η*, including proinflammatory cytokines interleukin (IL)-1*β*, IL-6, and TNF-*α*, as well as joint degradation factors such as MMP-9 and receptor activator of nuclear factor kappa-B ligand (RANKL). These factors can be blocked by preincubation with 14-3-3*η* antibodies [22].

At 6 months after anti-TNF therapy, there was a significant reduction of serum 14-3-3*η* levels in patients with early disease onset, suggesting a role in disease pathogenesis and potential as a therapeutic target for disease remission. Efforts are underway to understand the interplay between 14-3-3*η* and various chemokines/cytokines relevant to RA, as well as how specific therapies impact 14-3-3*η* serum levels [24]. Furthermore, studies are currently being conducted to investigate the therapeutic potential of targeting 14-3-3*η* in vivo using a collagen-induced arthritis model [22].

Regarding patients with established RA (group 2), disease activity was assessed clinically by DAS-28 (median 4.13) and ultrasonographic findings. Current data indicate a good correlation of MSUS with classical measures of clinical activity, and in some instances, MSUS appears to perform even better and has priority for the diagnosis of subclinical synovitis, which might help physicians in treatment planning [25]. The median synovial thickness in RA patients was 2.85 mm, 30% of patients showed grade 2 effusion, and 36.6% presented grade 2 Doppler activity.

We observed a highly significant positive correlation between serum 14-3-3*η* levels and DAS-28 and ultrasonographic findings in our patients of established disease (*p* < 0.001). Our study showed a significant positive correlation of serum 14-3-3*η* levels with ESR and CRP (*p* < 0.001 and 0.045, respectively). High CRP levels lead to the consideration that a progressive and erosive disease is present, so elevated serum 14-3-3*η* levels indicate disease progression [26]. Patients with lower serum levels of 14-3-3*η* at disease onset showed better clinical outcomes and a higher likelihood of achieving DAS remission in response to standard DMARDs. This was explained by an interplay that exists between 14-3-3*η* and TNF-*α*. More specifically, 14-3-3*η* can induce various inflammatory factors (including IL-6) in a dose-dependent fashion and may perpetuate the “cytokine storm” [24–27].

The median of RASS among RA patients was 70, and it was significantly correlated with 14-3-3*η* serum levels. The median MLS in RA patients was 34, and 31.7% of RA patients showed grade 2 bone erosion in ultrasonographic examination. Also, there was a significant positive correlation between 14-3-3*η* serum levels and radiological indicators of joint damage and disease severity.

14-3-3*η* is a potent, dose-dependent upregulation factor that perpetuates joint damage. Preliminary data from an analysis of 33 patients with RA revealed that median 14-3-3*η* levels were significantly higher in early RA patients with radiographic progression versus those of patients who did not progress (2.68 ng/ml vs. 0.09 ng/ml) [28]. A previous cohort study was conducted to demonstrate its role in disease prognosis, and 409 patients with early RA were recruited. Radiographic progression was assessed by the Sharp–van der Heijde score (SHS). 67% of patients were 14-3-3*η* positive, and in patients who progressed radiographically by year 5, there were significantly higher levels of 14-3-3*η* and RF, but not ACPA. Positive 14-3-3*η* status was significantly associated with radiographic progression at years 1, 3, and 5 with LR ranging from 3.8 to 6.8 [29].

Hirata et al. also reported this positive correlation, and the higher the 14-3-3*η* baseline levels were, the stronger the association with radiographic progression was [30]. Another study revealed significantly higher levels of 14-3-3*η* in patients who already had radiographic joint damage at the study baseline, as well as in those who developed progression by the end of the follow-up period. Monocytes from the THP-1 cell line were stimulated with 14-3-3*η* in a concentration similar to that of those found in the sera of RA patients. This resulted in the induction of proinflammatory cytokines IL-1*β*, IL-6, and TNF-*α*, as well as joint degradation factors such as MMP-9 and RANKL [22]. There was a significant positive correlation of 14-3-3*η* with RF and ACPA.

Forslund et al. [31] demonstrated the predictive value of ACPA for radiological changes. Other studies investigated that the ACCP antibody is better as a predictor of the disease course over 3 years [32, 33]. This supports the results of our study that the presence of ACCP and serum 14-3-3*η* is associated with joint destruction. Nevertheless, several independent multicenter studies are required to expand our understanding of the diagnostic, prognostic, and theragnostic applications of 14-3-3*η* biomarkers in RA for early therapeutic interventions and improving disease outcomes.

## 5. Conclusions

Conclusions: 14-3-3*η* has potential utility in the early diagnosis of RA with higher sensitivity and specificity compared to traditional diagnostic biomarkers. The addition of 14-3-3*η* as a novel biomarker to RF and ACCP is beneficial for early diagnosis of RA and early therapeutic intervention to reduce disease progression and structural damage. High serum levels of 14-3-3*η* in RA patients with established disease and its correlation with disease activity and severity reflect its role in disease pathogenesis, as well as its impact on disease progression. Thus, it warrants attention as a target for novel therapies. Furthermore, the significant reduction of serum 14-3-3*η* levels in patients with early RA at 6 months after anti-TNF therapy draws attention to its role in the pathogenesis and prognosis of the disease.

## Figures and Tables

**Table 1:** A comparison of the sensitivity of various RA biomarkers in patients with early and established disease. Serum 14-3-3 levels (ng/ml) were compared between the two RA groups, as well as the control and comparison of serum 14-3-3 levels before and after anti-TNF therapy in group I patients.

	Group I (early RA) (*n* = 80)	Group II (established RA) (*n* = 80)	*χ* ^2^:	^MC^ *p*
RF	61.7%	78.3%	3.968	0.0464^*∗*^
ACCP	68.3%	85%	4.658	0.0309^*∗*^
14-3-3*η*	86.7%	88.3%	0.0762	0.0783
*χ* ^2^: ^MC^*p*	17.023 < 0.001^*∗*^	3.854 0.146		

Serum 14-3-3*η* levels (ng/ml)	Group I (early RA) (*n* = 80)	Control	*t*	*P*
Min.-Max.	0.15–4.3	0.00–1.26	25.45	<0.001^*∗*^
Mean ± SD	2.34 ± 0.42	0.13 ± 0.56		

Serum 14-3-3*η* levels	Group II (established RA) (*n* = 80)	Control	*t*	*P*
Min.-Max.	0.17–5.1	0.00–1.26	37.88	<0.001^*∗*^
Mean ± SD	3.15 ± 0.26	0.13 ± 0.56		

Serum 14-3-3*η* in group I	Mean ± SD	*T*		*p*
Before treatment	2.34 ± 0.42	2.952		0.004^*∗*^
After treatment	2.03 ± 0.63			

MC: Monte Carlo; *χ*^2^: chi-square test.

**Table 2:** Clinical and radiological parameters of disease activity and clinical and radiological parameters of disease severity in patients with established RA.

Disease severity	Min.-Max.	Mean ± SD	Median
RASS	40.0–100.0	77.50 ± 18.03	70.0
Modified Larsen score MLS	20.0–64.0	34.30 ± 14.06	34.0
Disease activity score (DAS 28)	2.65–5.24	3.93 ± 1.35	4.13

*Ultrasonographic assessment (synovial thickness)*			
Min.-Max.		**1.95** mm–**4.80** mm	
Mean ± SD		**3.12** ± **1.24** mm	
Median		**2.85** mm	

Joint effusion		No.	%
Grade 0		**9**	**15**
Grade 1		**17**	**28.3**
Grade 2		**18**	**30.0**
Grade 3		**16**	**26.7**

*Doppler activity*			
Grade 0	**12**	**20.0**	
Grade 1	**16**	**26.7**	
Grade 2	**22**	**36.6**	
Grade 3	**10**	**16.7**	

Bone erosion (by US)	No.	%	
Grade 0	**9**	**15.0**	
Grade 1	**14**	**23.3**	
Grade 2	**19**	**31.7**	
Grade 3	**18**	**30.0**	

**Table 3:** Correlation between serum 14-3-3*η* levels and demographic, clinical, laboratory, radiological, and ultrasonographic data in established RA patients.

	*r*_*s*_	*H*	*P*
DAS 28 score	0.837		<0.001^*∗*^
ESR (mm/h)	**0.908**		**<0.001** ^*∗*^
CRP (mg/dl)	**0.382**		**0.045** ^*∗*^
RF (IU)	**0.767**		**0.009** ^*∗*^
ACCP (IU)	**0.667**		**0.001** ^*∗*^
RASS	**0.706**		**0.042** ^*∗*^
Modified Larsen score	**0.435**		**0.033** ^*∗*^
Synovial thickness	**0.676**		**<0.001** ^*∗*^
Effusion		**H** = **3.341**^*∗*^	**0.017** ^*∗*^
Erosions		**H** = **4.517**^*∗*^	**0.023** ^*∗*^
Doppler signal		**H** = **8.756**^*∗*^	**0.048** ^*∗*^

*H*: Kruskal–Wallis test.

**Table 4:** Agreement (sensitivity and specificity) for serum RF, ACCP, and 14-3-3*η* to predict early RA patients (vs. control).

	AUC	*p*	95% CI		Cutoff	Sensitivity	Specificity	PPV	NPV
			LL	UL					
RF	0.750	<0.001^*∗*^	0.646	0.830	>7.93	61.7	88.3	84.0	69.7
ACCP	0.817	<0.001^*∗*^	0.732	0.913	>21.20	68.3	95.0	93.1	75.0
14-3-3*η*	0.917	<0.001^*∗*^	0.854	0.998	>0.234	86.7	96.7	96.3	87.9

AUC: area under a curve, *p* value: probability value, CI: confidence interval, NPV: negative predictive value, PPV: positive predictive value, ^*∗*^: statistically significant at *p* ≤ 0.05

## Data Availability

The data used to support the findings of this study are included within the article.
